# Supplementary material for: Situating Wikipedia as a health information resource in various contexts: A scoping review
Source: PLoS One. 2020 Feb 18;15(2):e0228786. doi: 10.1371/journal.pone.0228786 (PMC7028268; doi:10.1371/journal.pone.0228786)
Supplement: S1 Appendix — (DOCX) [file pone.0228786.s001.docx]

# Appendix A: Search strategy and results

OVID Medline (June 3, 2019)

1. Encyclopedias as Topic/
2. Encyclopedia.ti,ab.
3. exp Internet/
4. internet.ti,ab.
5. online.ti,ab.
6. web.ti,ab.
7. world wide web.ti,ab.
8. Wikipedia.mp.
9. 1 or 2
10. or/3-8
11. 9 and 10
12. 8 or 11

Results: 476

OVID Embase (June 3, 2019)

1. encylopedia.ti,ab.
2. Internet/
3. internet.ti,ab.
4. online.ti,ab.
5. web.ti,ab.
6. world wide web.ti,ab.
7. Wikipedia.mp.
8. or/2-6
9. 1 and 8
10. 7 or 9

Results: 560

OVID AMED (June 3, 2019)

1. encyclopedia.ti,ab.
2. Internet/
3. internet.ti,ab.
4. online.ti,ab.
5. web.ti,ab.
6. world wide web.ti,ab.
7. Wikipedia.mp.
8. or/2-6
9. 1 and 8
10. 7 or 9

Results: 8

EBSCO CINAHL

1. TI Wikipedia
2. AB Wikipedia
3. 1 or 2

Results: 342

EBSCO Library and Information Science and Technology Abstracts (LISTA)

1. TI Wikipedia
2. AB Wikipedia
3. 1 or 2

Results: 1,380

EBSCO Library Literature and Information Science Full Text (Wilson Web)

1. TI Wikipedia
2. AB Wikipedia
3. 1 or 2

Results: 583

Web of Science

1. Wikipedia

Results: 4681

Total search results: 8,030
