# Supplementary material for: Situating Wikipedia as a health information resource in various contexts: A scoping review
Source: PLoS One. 2020 Feb 18;15(2):e0228786. doi: 10.1371/journal.pone.0228786 (PMC7028268; doi:10.1371/journal.pone.0228786)
Supplement: S2 Appendix — (DOCX) [file pone.0228786.s002.docx]

# Appendix B: General articles that aim to situate Wikipedia as a health information resource

**Table 1. General rticles that aim to situate Wikipedia as a health information resource**

| **Author(s)** | **Title** | **Source** | **Year** |
| --- | --- | --- | --- |
| Calabrese C, Anderton BN, Barnett GA. | Online Representations of “Genome Editing” Uncover Opportunities for Encouraging Engagement: A Semantic Network Analysis. | Science Communication | 2019 |
| Faric N, Potts HWW. | Motivations for contributing to health-related articles on Wikipedia: an interview study. | Journal of Medical Internet Research | 2014 |
| Heilman JM, Kemmann E, Bonert M, et al. | Wikipedia: a key tool for global public health promotion. | Journal of Medical Internet Research | 2011 |
| Heilman JM, West AG. | Wikipedia and Medicine: Quantifying Readership, Editors, and the Significance of Natural Language. | Journal of Medical Internet Research | 2015 |
| Laurent MR, Vickers TJ. | Seeking Health Information Online: Does Wikipedia Matter? | Journal of the American Medical Informatics Association | 2009 |
| Law MR, Mintzes B, Morgan SG. | The Sources and Popularity of Online Drug Information: An Analysis of Top Search Engine Results and Web Page Views | Annals of Pharmacotherapy | 2011 |
| Mesgari M, Okoli C, Mehdi M, Nielsen FÅ, Lanamäki A. | “The sum of all human knowledge”: A systematic review of scholarly research on the content of Wikipedia. | Journal of the Association for Information Science & Technology | 2015 |
| Okoli, C., Mehdi M, Mesgari, M., Nielson, F.A., Lanamäki, A. | Wikipedia in the eyes of its beholders: A systematic review of scholarly research on Wikipedia readers and readership. | Journal of the Association for Information Science & Technology | 2014 |
| Shafee T, Masukume G, Kipersztok L, Das D, Haggstrom M, Heilman J. | Evolution of Wikipedia’s medical content: past, present and future. | BMJ Journal of Epidemiology and Community Health | 2017 |
