# Supplementary material for: Situating Wikipedia as a health information resource in various contexts: A scoping review
Source: PLoS One. 2020 Feb 18;15(2):e0228786. doi: 10.1371/journal.pone.0228786 (PMC7028268; doi:10.1371/journal.pone.0228786)
Supplement: S3 Appendix — (DOCX) [file pone.0228786.s003.docx]

# Appendix C: Studies of Wikipedia content quality categorized by population of concern

**Table 2. Studies of the quality of Wikipedia for consumers or patients**

| **Author(s)** | **Title** | **Source** | **Year** |
| --- | --- | --- | --- |
| Biggs TC, Jayakody N, Best K, King EV. | Quality of online otolaryngology health information | The Journal of Laryngology & Otology | 2018 |
| Clauson KA, Polen HH, Boulos MNK, Dzenowagis JH. | Scope, completeness, and accuracy of drug information in Wikipedia. | Annals of Pharmacotherapy | 2008 |
| Czarnecka-Kujawa K, Abdalian R, Grover SC. | The quality of open access and open source internet material in gastroenterology: Is wikipedia appropriate for knowledge transfer to patients? | Gastroenterology | 2008 |
| James R. | WikiProject Medicine: Creating Credibility in Consumer Health. | Journal of Hospital Librarianship | 2016 |
| Koo M. | Complementary and Alternative Medicine on Wikipedia: Opportunities for Improvement. | Evidence-Based Complementary and Alternative Medicine | 2014 |
| Leithner A, Maurer-Ertl W, Glehr M, Friesenbichler J, Leithner K, Windhager R. | Wikipedia and osteosarcoma: a trustworthy patients’ information? | Journal of the American Medical Informatics Association | 2010 |
| Martin-Carreras T, Kahn CEJ. | Integrating Wikipedia Articles and Images into an Information Resource for Radiology Patients. | Journal of digital imaging | 2019 |
| Messner M, DiStaso MW, Yan Jin, Meganck S, Sherman S, Norton S. | Influencing public opinion from corn syrup to obesity: A longitudinal analysis of the references for nutritional entries on Wikipedia. | First Monday | 2014 |
| Modiri O, Guha D, Alotaibi NM, Ibrahim GM, Lipsman N, Fallah A. | Readability and quality of wikipedia pages on neurosurgical topics. | Clinical Neurology and Neurosurgery | 2018 |
| Nowrouzi B., Gohar B., Nowrouzi-Kia B., Garbaczewska M., Brewster K. | An examination of scope, completeness, credibility, and readability of health, medical, and nutritional information on the internet: A comparative study of Wikipedia, | Canadian Journal of Diabetes | 2015 |
| Qureishi A., Sharma A. | Can Wikipedia replace traditional patient information leaflets? Comparing the internet to official patient information resources in thyroid cancer. | European Surgery - Acta Chirurgica Austriaca | 2012 |
| Rajagopalan MS, Khanna VK, Leiter Y, et al. | Patient-oriented cancer information on the internet: a comparison of wikipedia and a professionally maintained database. | Journal of Clinical Oncology | 2010 |
| Simpson A, Le M, Malicka AN. | The Accuracy and Readability of Wikipedia Articles on Hearing Loss. | Journal of Consumer Health on the Internet | 2018 |
| Thomas GR, Eng L, de Wolff JF, Grover SC. | An Evaluation of Wikipedia as a Resource for Patient Education in Nephrology | Seminars in Dialysis | 2013 |
| Vydiswaran V.G., Mei Q., Hanauer D.A., Zheng K. | Mining consumer health vocabulary from community-generated text. | AMIA Annual Symposium proceedings. | 2014 |
| Watad A, Bragazzi NL, Brigo F, et al. | Readability of Wikipedia Pages on Autoimmune Disorders: Systematic Quantitative Assessment. | Journal of Medical Internet Research | 2017 |
| Weiner S.S., Horbacewicz J., Rasberry L., Bensinger-Brody Y. | Improving the Quality of Consumer Health Information on Wikipedia: Case Series. | Journal of medical Internet research | 2019 |

**Table 3. Studies of the quality of Wikipedia for students in health or medicine**

| **Author(s)** | **Title** | **Source** | **Year** |
| --- | --- | --- | --- |
| Antivalle M., Battellino M., Ditto M.C., et al. | Evaluation of wikipedia rheumatology articles as a learning resource for medical students | Annals of the Rheumatic Diseases | 2014 |
| Azer SA, AlSwaidan NM, Alshwairikh LA, AlShammari JM. | Accuracy and readability of cardiovascular entries on Wikipedia: are they reliable learning resources for medical students | BMJ Open | 2015 |
| Azer SA. | Is Wikipedia a Reliable Learning Resource for Medical Students? Evaluating Respiratory Topics. Adv Physiol Educ. | Advances in Physiology Education | 2015 |
| Azer SA. | Evaluation of gastroenterology and hepatology articles on Wikipedia: Are they suitable as learning resources for medical students? | European Journal of Gastroenterology & Hepatology | 2014 |
| Haigh CA. | Wikipedia as an evidence source for nursing and healthcare students. | Nurse Education Today | 2011 |
| Lavsa S.M., Corman S.L., Culley C.M., Pummer T.L. | Reliability of Wikipedia as a medication information source for pharmacy students. | Currents in Pharmacy Teaching and Learning | 2011 |
| Yacob M., Jetty P., Lotfi S. | The Wikipedia medical student: Comparing the quality of vascular surgery topics across two commonly used educational resources. | International Journal of Surgery | 2014 |
| Zhang Y., Lin Y.-H. | Writing a Wikipedia Article on Cultural Competence in Health Care. | Medical reference services quarterly | 2016 |

**Table 4. Studies of Wikipedia’s quality for professionals**

| **Author(s)** | **Title** | **Source** | **Year** |
| --- | --- | --- | --- |
| Bould MD, Hladkowicz ES, Pigford A-AE, et al | References that anyone can edit: review of Wikipedia citations in peer reviewed health science literature. | BMJ | 2014 |
| Devgan L, Powe N, Blakey B, Makary M. | Wiki-Surgery? Internal validity of Wikipedia as a medical and surgical reference. | Journal of the American College of Surgeons | 2007 |
| Hasty RT, Garbalosa RC, Barbato VA, et al. | Wikipedia vs peer-reviewed medical literature for information about the 10 most costly medical conditions. | The Journal of the American Osteopathic Association | 2014 |
| Morata T., Lum M. | Partnerships to expand occupational safety and health content in Wikipedia. | Occupational and Environmental Medicine | 2018 |
| Park E., Masupe T., Joseph J., et al. | Information needs of Botswana health care workers and perceptions of Wikipedia. | International Journal of Medical Informatics | 2016 |
| Reilly T, Jackson W, Berger V, Candelario D. | Accuracy and completeness of drug information in Wikipedia medication monographs. | Journal of the American Pharmacists Association | 2017 |

**Table 5. Studies of Wikipedia content quality where no specific population is considered**

| **Author(s)** | **Title** | **Source** | **Year** |
| --- | --- | --- | --- |
| Altmann U. | Representation of medical informatics in the wikipedia and its perspectives. | Studies in health technology and informatics | 2005 |
| Ayes KB, Bardsley CH, Jantz JM, Frederick WA | Wikipedia information for toxicologic emergencies involving household products, plants and envenomations: How reliable is it? | Clinical Toxicology | 2011 |
| Ayes KB, Bardsley CH. | Wikipedia Information for Toxicologic Emergencies: How Reliable Is It? | Clinical Toxicology | 2010 |
| Cozza V, Petrocchi M, Spognardi A. A | Matter of Words: NLP for Quality Evaluation of Wikipedia Medical Articles. | Lecture Notes in Computer Science | 2016 |
| Hillard J.R., Laird-Fick H.S., Chennamaneni V., Kavuturu S., Nassiri R.M. | Helicobacter and Wikipedia. | Helicobacter. Supp 1. | 2015 |
| Holtz P, Fetahu B, Kimmerle J. | Effects of Contributor Experience on the Quality of Health-Related Wikipedia Articles. | Journal of Medical Internet Research | 2018 |
| Hunter JA, Lee T, Persaud N. | A comparison of the content and primary literature support for online medication information provided by Lexicomp and Wikipedia. | Journal of the Medical Library Association: JMLA | 2018 |
| Koppen L, Phillips J, Papageorgiou R. | Analysis of reference sources used in drug-related Wikipedia articles. | Journal of the Medical Library Association: JMLA | 2015 |
| Pfundner A, Schönberg T, Horn J, Boyce RD, Samwald M. | Utilizing the Wikidata System to Improve the Quality of Medical Content in Wikipedia in Diverse Languages: A Pilot Study | Journal of Medical Internet Research | 2015 |
| Phillips J, Lam C, Palmisano L. | Analysis of the accuracy and readability of herbal supplement information on Wikipedia. | Journal of the American Pharmacists Association | 2014 |
| Reavley NJ, Mackinnon AJ, Morgan AJ, et al. | Quality of information sources about mental disorders: a comparison of Wikipedia with centrally controlled web and printed sources. | Psychological medicine | 2012 |
| Sanz-Valero J, Cabrera-Hernandez L, Wanden-Berghe C, Culebras-Fernandez JM | The popularization of food and nutritional sciences: Wikipedia versus a general encyclopedia. | Clinical Nutrition | 2013 |
| Sanz-Valero J, Wanden-Berghe C, Guardiola-Wanden-Berghe R. | Nutrition and metabolism in Wikipedia: Presence and adequacy of English and Spanish terminology | Clinical Nutrition | 2012 |
| Skancke KH, Svendsen K. | Wikipedia and medicines: who edits medicine articles on the English Wikipedia? | International Journal of Clinical Pharmacy | 2017 |
| Stankus T, Spiegel SE. Wikipedia, | Scholarpedia, and References to Journals in the Brain and Behavioral Sciences: A Comparison of Cited Sources and Recommended Readings in Matching Free Online Encyclopedia Entries | Science & Technology Libraries | 2010 |
| Temple NJ, Fraser J. | How Accurate Are Wikipedia Articles in Health, Nutrition, and Medicine? | Canadian Journal of Information and Library Science | 2014 |
