# Supplementary material for: Situating Wikipedia as a health information resource in various contexts: A scoping review
Source: PLoS One. 2020 Feb 18;15(2):e0228786. doi: 10.1371/journal.pone.0228786 (PMC7028268; doi:10.1371/journal.pone.0228786)
Supplement: S4 Appendix — (DOCX) [file pone.0228786.s004.docx]

# Appendix D: Studies of Wikipedia’s utility in health education, categorized by education type

**Table 6. Studies of Wikipedia’s utility in graduate or undergraduate health or medical education**

| **Author(s)** | **Title** | **Source** | **Year** |
| --- | --- | --- | --- |
| Apollonio D.E., Broyde K., Azzam A., De Guia M., Heilman J., Brock T. | Pharmacy students can improve access to quality medicines information by editing Wikipedia articles. | BMC medical education | 2018 |
| Azzam A., Bresler D., Leon A., et al. | Why Medical Schools Should Embrace Wikipedia: Final-Year Medical Student Contributions to Wikipedia Articles for Academic Credit at One School. | Academic Medicine | 2017 |
| Burdo JR | Wikipedia neuroscience stub editing in an introductory undergraduate neuroscience course. | Journal of undergraduate neuroscience education: JUNE | 2012 |
| Herbert VG, Frings A, Rehatschek H, Richard G, Leithner A. | Wikipedia--challenges and new horizons in enhancing medical education. | BMC Medical Education | 2015 |
| Infeld DL, Adams WC. | Using the Internet for Gerontology Education: Assessing and Improving Wikipedia. | Educational Gerontology | 2013 |
| Judd T, Kennedy G. | Expediency-Based Practice? Medical Students’ Reliance on Google and Wikipedia for Biomedical Inquiries. | British Journal of Educational Technology | 2011 |
| Kim JY, Gudewicz TM, Dighe AS, Gilbertson JR. | The pathology informatics curriculum wiki: Harnessing the power of user-generated conten | Journal of pathology informatics | 2010 |
| Murray H., Walker M., Maggio L., Dawson J. | Wikipedia medical page editing as a platform to teach evidence-based medicine. | BMJ Evidence-Based Medicine | 2018 |
| Scaffidi MA, Khan R, Wang C, Keren D, Tsui C, Garg A, et al. | Comparison of the impact of Wikipedia, UpToDate, and a Digital Textbook on Short-Term Knowledge Acquisition Among Medical Students: Randomized Controlled Trial of Three Web-Based Resources. | Journal of Medical Interent Research | 2017 |
| Schweitzer NJ. | Wikipedia and psychology: Coverage of concepts and its use by undergraduate students. | Teaching of Psychology | 2008 |
| Shane-Simpson C, Che E, Brooks PJ. | Giving Psychology Away: Implementation of Wikipedia Editing in an Introductory Human Development Course. | Psychology Learning and Teaching: PLAT | 2016 |
| Walker M., Murray H., Dawson J., Maggio L. | Wikipedia culture and usage: A survey of first year medical students to determine barriers and facilitators. | BMJ Evidence-Based Medicine | 2018 |

**Table 7. Studies of the utility of Wikipedia in professional or continuing education**

| **Author(s)** | **Title** | **Source** | **Year** |
| --- | --- | --- | --- |
| Good B.M., Clarke E.L., Loguercio S., Su A.I. | Building a biomedical semantic network in Wikipedia with Semantic Wiki Links | Database: the journal of biological databases and curation | 2012 |
| Good BM, Howe DG, Lin SM, Kibbe WA, Su AI. | Mining the Gene Wiki for functional genomic knowledge. | BMC genomics | 2011 |
| Huss JW 3rd, Lindenbaum P, Martone M, et al. | The Gene Wiki: community intelligence applied to human gene annotation. | Nucleic acids research | 2010 |
| Lieberthal RD, Leon J. | Engaging Health Professionals in Health Economics: A Human Capital Informed Approach for Adults Learning Online. | Journal of Economic Education | 2015 |
| Rossler B., Holldack H., Schebesta K. | Influence of wikipedia and other web resources on acute and critical care decisions. A web-based survey. | Intensive Care Medicine Experimental | 2015 |
