# Supplementary material for: Situating Wikipedia as a health information resource in various contexts: A scoping review
Source: PLoS One. 2020 Feb 18;15(2):e0228786. doi: 10.1371/journal.pone.0228786 (PMC7028268; doi:10.1371/journal.pone.0228786)
Supplement: S5 Appendix — (DOCX) [file pone.0228786.s005.docx]

# Appendix E: Studies of Wikipedia’s utility in health-related research, categorized by field

**Table 8. Studies of Wikipedia’s utility in health-related research in the field of human information practices**

| **Author(s)** | **Title** | **Source** | **Year** |
| --- | --- | --- | --- |
| Brigo F, Erro R. | Why do people google movement disorders? An infodemiological study of information seeking behaviors. | Neurological Sciences | 2016 |
| Brigo F, Igwe SC, Nardone R, Lochner P, Tezzon F, Otte WM. | Wikipedia and neurological disorders. | Neuroepidemiology | 2014 |
| Brigo F., Igwe S.C., Otte W.M., Tezzon F., Lochner P. | Information seeking behavior for neurological disorders: An infodemiological study of wikipedia article traffic | Neuroepidemiology | 2014 |

**Table 9. Studies of Wikipedia’s utility as a data source in the field of bibliometrics**

| **Author(s)** | **Title** | **Source** | **Year** |
| --- | --- | --- | --- |
| Evans P, Krauthammer M. | Exploring the use of social media to measure journal article impact. | AMIA Annual Symposium proceedings | 2011 |
| Jemielniak D., Masukume G., Wilamowski M. | The Most Influential Medical Journals According to Wikipedia: Quantitative Analysis | Journal of medical Internet research | 2019 |
| Kint M., Batty P., Peters M., Hart D. | Wikipedia dominance in simulated online searching for hemophilia specific patient information | Haemophilia | 2014 |
| Kissner M, Troia L, Bexfield A, Mahbuba R, Coover R. | Evaluating the relationship between publications and Wikipedia citations for a cohort of oral anticoagulants. | Current Medical Research and Opinion | 2019 |
| Maggio LA, Willinsky JM, Steinberg RM, Mietchen D, Wass JL, Dong T. | Wikipedia as a gateway to biomedical research: The relative distribution and use of citations in the English Wikipedia. | PLoS ONE | 2017 |

**Table 10. Studies of Wikipedia’s utility as a data source in the field of epidemiology**

| **Author(s)** | **Title** | **Soure** | **Year** |
| --- | --- | --- | --- |
| Dzogang F, Lansdall-Welfare T, Cristianini N. | Seasonal Fluctuations in Collective Mood Revealed by Wikipedia Searches and Twitter Posts. | 2016 IEEE 16^th^ International Conference on Data Mining Workshops | 2016 |
| Generous N, Fairchild G, Deshpande A, Del Valle SY, Priedhorsky R. | Global Disease Monitoring and Forecasting with Wikipedia. | PloS Computational Biology | 2014 |
| McIver DJ, Brownstein JS. | Wikipedia Usage Estimates Prevalence of Influenza-Like Illness in the United States in Near Real-Time. | PloS  Computational Biology | 2014 |
| Priedhorsky R, Osthus D, Daughton AR, et al. | Measuring Global Disease with Wikipedia: Success, Failure, and a Research Agenda. | CSCW : proceedings of the Conference on Computer-Supported Cooperative Work. | 2017 |
| Qiu R, Hadzikadic M, Yu S, Yao L. | Estimating disease burden using Internet data. | Lecture Notes in Artificial Intelligence | 2017 |
| Sharpe JD, Hopkins RS, Cook RL, Striley CW. | Evaluating Google, Twitter, and Wikipedia as Tools for Influenza Surveillance Using Bayesian Change Point Analysis: A Comparative Analysis. | JMIR public health and surveillance | 2016 |
| Tausczik Y, Faasse K, Pennebaker JW, Petrie KJ. | Public anxiety and information seeking following the H1N1 outbreak: blogs, newspaper articles, and Wikipedia visits. | Health Communication | 2012 |
| Zimmer C., Leuba S.I., Yaesoubi R., Cohen T. | Use of daily Internet search query data improves real-time projections of influenza epidemics. | Journal of the Royal Society Interface | 2017 |
