# Supplementary material for: Situating Wikipedia as a health information resource in various contexts: A scoping review
Source: PLoS One. 2020 Feb 18;15(2):e0228786. doi: 10.1371/journal.pone.0228786 (PMC7028268; doi:10.1371/journal.pone.0228786)
Supplement: Attachment — Submitted filename: review PLOS Manuscript Number PONE-D-19-29040.pdf [file pone.0228786.s006.pdf]

I am Lane Rasberry and I develop medical information on Wikipedia.
https://en.wikipedia.org/wiki/User:Bluerasberry

I copy lines from the paper then comment after.

Excellent draft - if you changed nothing then it is fit for publication. Suggestions below are optional for you to use.

37 “Imagine a world in which every single person has free access to the sum of all medical 38 knowledge” (1). This is what WikiProject Medicine, a community of editors who develop 39 the health care content in Wikipedia (2),

Some clarification here - omit the link to Wordpress, your citation #1. This is not maintained and is the same as the #2 citation
https://meta.wikimedia.org/wiki/Wiki_Project_Med

There are two projects. WikiProject Medicine is the on-wiki community. WikiProjects are an established custom. There is no legal entity for this.

Wiki Project Med, also called Wiki Project Med Foundation, is a New York state based 501(c)3 organization with international membership and which represents Wikipedia contributors who want affiliation with a legal organization.

31-33
Several questions have not been asked within the literature that are instrumental in understanding Wikipedia’s role as a health information resource.

Examining the research literature will not lead to understanding Wikipedia. I wish it were, but literature review is no path to this. The claim that the literature is instrumental seems misguided.

41-42
than any other health information 42 resource online

"than any other single health information resource"
I realize that we in Wikipedia do not track non-digital media because it is not possible, but Wikipedia is a continually living 20 year global resource. It competes against things like personal doctor visits.

I think you went to the Google / Facebook / Microsoft event at the WikiConference. I have less understanding of Google, but I think they said that 15% of user queries are health related. I feel

increasingly comfortable calling Wikipedia most popular without qualifiers like "online", "new media", etc.

45
However, a paradigm45 shifting study conducted by Nature found that Wikipedia and Encyclopedia Britannica

This was in 2005, we have high school students born after this who use Wikipedia routinely. Since 2005 there has been one human generation of development passed and several technological generations. For example, in 2005 no one thought Facebook would become popular. . There has been no big challenge of Wikipedia since then. There have been various small challenges.

You know the literature. My point in this is just that the Nature study is old and the bigger news is that no one ever challenged it.

51
that editing Wikipedia is influenced predominantly by an 51 editor's inherent values

yes correct

56 the pages of Wikipedia is a community of (mostly) altruistic editors motivated

Yes, everyone includes altruism in their motivations. We have no long-term examples of anyone editing Wikipedia for pay and having community integration or positive ongoing relationships. There are only a few identified paid editors, maybe 10-15 who make themselves known in the entire world.

59 Today Wikipedia is the 7th most frequently accessed web site in the United States and 60 Canada(7).

Since 2005 Wikipedia has been an Alexa top 10 website. There is no source to cite for this. Evidence becomes stronger after 2007 and stronger again after 2012, but no one has published this simple reporting. If you feel bold enough you could say "top ten website since at least 2014" and cite Heilman 2015
https://www.ncbi.nlm.nih.gov/pmc/articles/PMC4376174/
the strength of the claim in the source is weak but the statement is accurate

60 For perspective, English Wikipedia alone averages approximately 830 million 61 unique visits per month globally(8).

Right. WMF says 1 billion uniques / year, and that "billions" (meaning 2) have visited. 2 billion is 25% of world population.
https://annual.wikimedia.org/2017/

74 developed and executed a search strategy

FYI - I am unable to comment on best library practices. I assume you did what is normal and orthodox here.

I wish you could use WikiCite, Wikipedia's own data corpus for academic literature. I would not want to put you out of your way but if it were easy for you to name drop WikiCite and its search function Scholia then I would appreciate it. I work on both of these projects. Here is the Scholia profile of the Wikicite corpus of source metadata for academic papers tagged with subject "health information on Wikipedia". https://tools.wmflabs.org/scholia/topic/Q16235120
Also, I wish to reconcile the set of papers you examined to Wikicite, tagging everything you found as "health information on Wikipedia" in case I missed anything.

Wikicite has 90 papers, you found 89, and I wonder what the difference is.

98 treatment of Wikipedia's health content was insufficient to merit inclusion. These articles 6 99 will be reviewed separately in an additional study of the literature that specifically explores 100 online health information seeking behaviour and Wikipedia's role within it.

I get anxious seeing promises like this made because it can be hard to identify the later paper if it exists. I sort of wish this promise could be omitted.

108 Fig 1. PRISMA Flow Diagram

love PRISMA
https://en.wikipedia.org/wiki/Preferred_Reporting_Items_for_Systematic_Reviews_and_Meta-Analyses

110 In 2018, a study based on a sample of 100,000 articles indexed in CrossRef, a database that 111 indexes al

This bit about OA is interesting but this is the start of the results section, and I was hoping for a strong start about Wikipedia. The OA information could come later as it is more incidental.

117 openness and the broad dissemination of information. It is encouraging to see evidence of 118 similar values between those investigating Wikipedia and the organization itself.

Can you clarify what "the organization" is? Perhaps rephrase?

124 literature about Wikipedia is illustrated by a noticeable peak in 2014 (Fig 3), the same year 125 the only two known systematic reviews of Wikipedia scholarship were published.

Wait - can you cite the reviews to which you are referring?

125 the only two known systematic reviews of Wikipedia scholarship were published. In 2015, 126 Nature reported its findings that suggested Wikipedia was more or less equal in accuracy 127 compared to Encyclopedia Britannica

Seems like an error! Not 2015, 2005!
https://www.nature.com/articles/438900a
I am not aware of a 2015 paper!

123 -129
perhaps, but is this a confusion about the date of the nature paper? If so then revise or strike this.

134 1. Articles with a general focus on situating Wikipedia as a health information resource 135 with no specific context as a major focus (e.g for consumers, professionals, or 136 students);

It seems extraneous to qualify this when this is the only category of this sort. Why not just say "Articles with a general focus on situating Wikipedia as a health information resource", then later explain this additional detail that you sought no specific audience targeting?

156 dominate the conversation. 53% (n=47) of the articles included in this study measure

Is "measure" the correct term? I would have expected "report" or "make claims upon", but I think most studies do not do a measurement in a quantitative way.

160 currency, comprehensiveness, completeness of coverage, number of references, and 161 currency.

currency twice
also I know that Wikipedia emphasizes number of references, but I am not aware of other sources strongly valuing citations in medical information like Wikipedia does.

181 resource demonstrate this by references its frequency of use and some advocate for the 182 medical community to contribute to Wikipedia’s medical content. E

something unclear here - "references" to "referencing", perhaps

182 medical community to contribute to Wikipedia’s medical content. Evidence has been 183 collected from the Pew Research Centre

This confuses me a bit. Active voice could help -

The Pew Reseach Center collected evidence...

218 same topic s(17). This is study is seminal positioning Wikipedia’s prominence in the 219 existing complement of online health information resources.

rephrase

This study positions Wikipedia as prominent among the existing...

227 gastroenterology (18), nephrology (19), and some cancers (20–22). These assessments 228 assess

"assessments assess" to "studies assess" or similar

229 findings in relation to the public consumer or patient. Of those that include results – some 230 conference proceedings do not – they generally agree that Wikipedia is suitable for patients 231 (18,19,22) and a 2010 study find

Cut this into multiple sentences. The hyphen conjuctions etc is too much.

232 the preferred one (21). Unfortunately, these assessments of suitability for patients are no 233 longer likely to be relevant with publication dates ranging between 2008 and 2013.

The situation is mixed. Many articles require updating and some do not.

236 (2017) investigation into who edits Wikipedia’s medicines content

"medicines" to medical, as this not about drugs specifically

242 information in Wikipedia about a specific health speciality. These include autoimmune
Yes, and they make it difficult to generalize anything.

248 Two distinctive studies describe leveraging Wikipedia’s content in order to improve 249 patient care and this is perhaps the most exciting finding of this review.

I do not follow why you think these are the most exciting. Can you explain more?

251 now integrated with Wikipedia to pull radiology images from the WikiMedia Commons into

It is stylized "Wikimedia Commons"

252 their database (46). Another study describes how it used Wikipedia to mine consumer 253 health vocabulary by finding synonyms for medical terminology

Excavating the mother lode
https://www.sciencedirect.com/science/article/pii/S0306457316303004

This talks about medicine and the people behind this are wiki medicine people. Not sure if you detected this one as it does not have Wikipedia or medicine in the title. The paper was bold enough to identify Wikipedia as the mother lode but regrettably did not say Wikipedia in the title. They really are sitting on a big idea.

That was out of scope for you but confirms the application.

262 the findings cannot be generalized (48). Interestingly this study also finds that of the 263 participants, 65% did not know how to revise Wikipedia when they encountered an error.

prefer "did not know that Wikipedia invites them to edit text to revise errors which they encounter"

277 studies, and the findings vary from one study to another. Some conclude Wikipedia is 278 suitable for medical students(53,57), but most conclude it is not(53–56,58,59).

yeah, I wish more studies would compare Wikipedia's suitability to any other specific thing instead of comparing it to the ideal of perfection.

284 Further, Wikipedia's medical content is indicated as valuable simply because it is freely 285 available and, through a relationship with telecommunications companies, remains 286 accessible when internet access is lost and other online information sources are rendered 287 unavailable (60)..

That project, Wikipedia Zero, is sunsetted. Regardless of publication and evaluation, we in the Wikipedia editing and outreach community never became aware of any cases of use which resulted in anyone contacting anyone in our social network. It was kind of an expensive outsider project which never had Wikipedia community collaboration in an apparent way.

The community effort, named as a flagship Wikimedia Foundaiton accomplishment in their 2018 annual report, is Internet in a Box. I and lots of WikiProject Medicine people contribute to this.
https://meta.wikimedia.org/wiki/Internet-in-a-Box

I wish you could omit this text just because that Wikipedia Zero project is over, it never matured, it had more presence in publication than in Wikipedia community engagement, and it was kind of a project on paper which had thin ties to any reality. I do not want to criticize it because it could be revived, good people contributed to it, and is still a good idea but we do not have much to show for this.

314 that while Wikipedia does well to remain current (27,42,44), the readability of its medical 315 content is too low to accommodate the very people it stands to benefit most. The lowest 316 reading level reported is around ninth grade (26,31,41)

clarity here please

you use the term "low" in two different senses. One sense is "low readability", which I think means "high complexity", and the other sense is "low reading level", which means "low complexity".

perhaps

that while Wikipedia does well to remain current (27,42,44), its medical content uses technical terms which result in readability which is too low accommodate the very people it stands to benefit most. The most accessible reading level reported is around ninth grade (26,31,41)

325 methodological approaches assess Wikipedia in comparison to a resource with which 326 significant differences should be expected, such as subscription-based reference tools 327 designed for practitioners or experts (28,31,36,37), or websites managed by leading 328 organizations on the topic (42)

I think it is fair to compare Wikipedia with leading resources, regardless of whether they claim to have good subscription funding or target expects. The dubious methodologies include things like setting comparisons where Wikipedia does not try to be competitive, like taking points off Wikipedia for not including dose information or professional practice information. Wikipedia competes as reference information and should be compared on that basis.

328 organizations on the topic (42). More appropriate comparisons are drawn between other 329 open, free tertiary resources (27) or, alternatively, a validated instrument, such as 330 DISCERN, is used to quantitatively assess Wikipedia articles (34,35,43,63)

two kinds of comparisons - reference information quality is one issue, and accessibility is another issue.

For quality I want Wikipedia compared against the best competing resource for any topic. Along with that comparison, there can also be a comparison of accessibility.

Wikipedia could then also be compared against the best available free resource, but I see little value in that because I expect that nothing would come close to Wikipedia's quality. Also any other available free resource is almost certainly negligible traffic and therefore inaccessible.

339 question(64) while another neglects to acknowledge a severe mismatch between the 340 sample studied and the results of the study(65)

intentional mismatch is the norm and it is not fair. I do not know what everyone gets out of obviously ill designed wiki bashing reports.

347 Any attempt to generalize findings in order to generate a holistic understanding of the 348 quality of the entirety Wikipedia’s medical content is an insurmountable task.

specific the methodology which you imagine

I admit that we do not have a holistic understanding but also no one has tried. I think if we had modest funding to do an evaluation then we could do well to get a good general understanding. There are several possible strategies to attempt and realistically there has hardly been any attempt.

I am much more hopeful about this.

351 more complete than others. Wikipedia is also fluid. While it is prudent to describe 352 Wikipedia as a tertiary information resource, it is not equivalent to a published 353 encyclopedia. Not only is it incomplete, it is also dynamic: evolving, and expanding; thus

It has been 20 years. Wikipedia is the golden standard for what defines a published encyclopedia. Saying that it is "not equivalent to a published encyclopedia" is odd in the context that Wikipedia is known as an encyclopedia and gets more human hours of consultation than all other encyclopedias put together. At some point it becomes necessary to state what one expects of an encyclopedia and what Wikipedia is.

357 Wikipedia’s content, it is a challenge to understand their value. Studies that measure the 358 quality of Wikipedia’s health information seem to be asking the wrong question. Further, 359 recommendations for or against the use of Wikipedia in any context do not take into 360 consideration Wikipedia’s utility.

Be direct, what is the wrong question, and what is the right question

368 The first known for-credit course at a medical school for editing Wikipedia was offered by 369 University of California, San Francisco in 2013 (50),

"The first documented record of a for credit course at a medical school"

I led a Wikipedia editing series at the University of Washington medical school in 2011. When I did that there were a few others doing similar things. When that 2013 program began I trained Amin Azzam based on the culture of practice for medical editing in schools which already existed for this.

I guess that I am realizing that I need to write up some history because many people at many institutions contributed greatly, and I want the origin story to be Wikipedia-style normal community collaboration and not one person's new idea.

369 University of California, San Francisco in 2013 (50), but the first reported use of Wikipedia 370 in a course, as a single assignment, is from 2011 and it required students to edit 371 neuroscience stub articles (71)

You might mean "medical course". There are probably earlier records of medical school course editing on Wikipedia, but not in academic publishing. Likewise there are earlier records of class editing. United States Public Policy in 2010 was the first big classroom initiative.
https://en.wikipedia.org/wiki/Wikipedia:WikiProject_United_States_Public_Policy
even this followed earlier classroom experiments.

372 education courses has crossed a number of health disciplines. Pharmacy students have 373 been asked to write content in Wikipedia as an alternative to composing pharmaceutical 374 drug monographs (70) and a gerontology instructor identified gaps in Wikipedia's content 375 on aging and assigned students the task of using their term papers to edit it (72).

Here is my own study about classroom use. If you are looking for examples of classroom engagement then this program is in its 5th year and of course this paper is Wikipedia-values oriented.

Improving the Quality of Consumer Health Information on Wikipedia: Case Series
https://www.jmir.org/2019/3/e12450/

394 to UpToDate and a digital textbook

check your consistent stylization
UpToDate

411 GeneWiki, a collection of Wik

Great project to mention'

441 of evidence(82,83). There is a notable trend in the assertion that one major value of 442 Wikipedia is its curated list of published articles, m

not sure what this means, clari fy please

458 context of research focused on Wikipedia's potential role as a data source in 459 epidemiological research. Generous, e

you mentioned the Ebola study above. Maybe that is better placed here? The context of that was that the WHO did not have expertise to translate Ebola text into languages of Africa, but Wikipedia did have community of translators, and a platform to accept this language content, and the means of distribution for this information. If you want an epidemiological example then that ebola incident is the one the media picked up.

470 As promising as the research into the potential utility of Wikipedia in epidemiology may be, 471 one major limitation of using Wikipedia for outbreak prediction is often touted as one of 472 Wikipedia's strengths: a

This is a misunderstanding. Google will not talk to university researchers. Google is inhuman and like aliens far away. Wikipedia is a human project and of course we have the data to emulate Zeiger's Google Flu Trends project. Typical researchers can also ask for some private IP address data. Wikipedia does not share this data lightly, but whereas Google almost certainly would not share data with less than a ~300 million investment in this, the buy in for access to Wikipedia's data is ~1 million and commitment to act like a human while having conversations in public with the wiki community. Of course we want to do this project, and the barrier is not on the Wikipedia side of this.

489 than other free online health information sources, such as Medline Plus, and its pages 490 regularly rank highly in Google search results,

you could say Bing and DuckDuckGo, or you could say search engines. Google is not quite universal. Russia and Korea use other search engines, and lots of other countries do too. China is moving onto the world stage and probably will compete against Google in the developing world with their search engines eventually. If you generalize this paper has some more life and citability in the future.

499 would need to be assessed in order to properly evaluate its medical content holistically 500 often renders these types of evaluations irrelevant.

Again, we have tried nothing and failed. The cost of trying something bit would be the low US$100ks.

512 within the context of health is still in the early stages of its development. Several questions 513 have not yet been asked within the literature that are instrumental in understanding 514 Wikipedia's role as a health information resource and various contexts: as a patient or 515 consumer health information resource, as a resource for medical or health students, as a 516 resource for professional in health and medicine, and as a data resource for researchers.

can you rephrase? this is long and blocky and I cannot readily see what questions you want asked and answered.

520 explaining the technicalities of genome editing and do not necessarily focus on other hotly 521 debated conversations around the topic, such as ethics (9).

Obviously the issue is that the genetics people import structured data at scale, which is relatively easy, whereas for ethics we require prose which is more difficult.

525 data provides evidence that Wikipedia is accessed frequently, this data is insufficient to 526 understand whether its content is actually used by the consumer.

I know why you say this because everyone says this but it is totally bunk. No one holds other communication channels to this standard. Everyone assumes that doctor's office pamphlets and billboards and online patient guides and apps andWe have no reasonable evidence of how consumers use other sources. Suddenly when Wikipedia appears among other communication resources somehow people question whether users get impact out of Wikipedia while taking for granted that they get impact from other communication channels. It is not reasonable and it indicates a bias against Wikipedia.

You are not citing sources on this so if this is your own musing, I would prefer that this be phrased like this.

There is evidence that Wikipedia is more popular than other channels, but some critics dismiss this by saying that the value of measurable user attention to Wikipedia is less than the value of user attention to other sources. It would be useful to have a system to assign impact value consistently to Wikipedia and other outreach channels so that their usefulness could be compared.

Figure 2

Can you label this more explicity? Perhaps you do this in the text of the article. Just looking at the image I became lost understanding the vertical access labels.

Figure 4

You might have already described this but I missed the place. Was the classification obvious or did you use a non-obvious sorting system?
